# Plasma homocysteine levels and intracranial plaque characteristics: association and clinical relevance in ischemic stroke

**DOI:** 10.1186/s12883-018-1203-4

**Published:** 2018-12-06

**Authors:** Shan shan Lu, Jun Xie, Chun qiu Su, Song Ge, Hai bin Shi, Xun ning Hong

**Affiliations:** 1 Department of Radiology, The First Affiliated Hospital of Nanjing Medical University, No.300 Guangzhou Road, Gulou district, Nanjing, 210029 Jiangsu Province China; 2 Department of Neurology, The First Affiliated Hospital of Nanjing Medical University, Nanjing, 210029 Jiangsu Province China

**Keywords:** Homocysteine, Atherosclerosis, Magnetic resonance imaging, Ischemic stroke

## Abstract

**Background:**

Elevated plasma homocysteine (Hcy) is an independent risk factor for ischemic stroke. This study aimed to evaluate the association between Hcy levels and intracranial plaque characteristics and to investigate their clinical relevance in ischemic stroke.

**Methods:**

Ninety-four patients with intracranial atherosclerosis (ICAS) were enrolled. Plasma Hcy levels were measured. Intracranial plaque characteristics including plaque enhancement, stenosis ratio, T2 and T1 hyperintense components were assessed on high-resolution magnetic resonance imaging. Logistic regression model was constructed to analyze the association between high Hcy levels and plaque characteristics, and their synergistic effects to predict the likelihood for ischemic stroke, while adjusting for demographics and traditional atherosclerotic risk factors.

**Results:**

Elevated Hcy level was associated with strong plaque enhancement independently of age, sex, serum creatinine levels and other atherosclerotic risk factors ((*P* < 0.001, OR 6.00, 95% confidence interval [CI] 2.28–15.74). Both strong plaque enhancement (*P* = 0.026, OR 5.63, 95% CI 1.23–25.81) and high Hcy level (*P* = 0.018, OR 6.20, 95% CI 1.36–28.26) were correlated with acute ischemic stroke. The combination of them strengthened the ability to stratify the likelihood for ischemic stroke, with an improved area under the receiver operating characteristic curve (AUC) of 0.871, significantly higher than that of strong plaque enhancement (0.755) and high Hcy level (0.715) alone (*P* < 0.05 for both).

**Conclusions:**

High Hcy level appears to have association with intracranial strong plaque enhancement. The combined assessment of plasma Hcy levels and plaque enhancement may improve ischemic stroke risk stratification.

## Background

Acute ischemic stroke is the most common cerebrovascular disease and is one of the leading causes of death and long-term disability through the world [1]. Large artery atherosclerosis is a major cause of ischemic stroke and can be divided into intracranial atherosclerosis (ICAS) and extracranial atherosclerosis [2]. Recently, studies have reported that the prevalence of ICAS has racial and ethnic differences, with a greater incidence in Asian people [3]. A study by Cao et al. exhibited significantly greater plaque burden of middle cerebral artery (MCA) as compared to ipsilateral extracranial carotid artery and is more strongly associated with ischemia severity [4]. Bos et al. established ICAS as a major risk factor for stroke and suggested that its contribution to the proportion of all strokes may be greater than that of extracranial atherosclerosis [5]. Similarly, a retrospective study by Wong et al. also demonstrate that although extracranial atherosclerosis may be a more common lesion in whites in Europe and America, ICAS is more common in Asian patients [6].

Elevated plasma homocysteine (Hcy) levels have been indicated as an independent risk factor for ischemic stroke [7, 8]. High Hcy levels may be associated with endothelial injury and vascular inflammation, leading to the development of atherogenesis [9]. Studies have revealed that high Hcy levels are associated with advanced carotid plaque and greater incidence of acute ischemic stroke in patients with carotid artery plaques [7, 10–13]. However, most of the previous findings were derived from carotid artery, presumably due to the technical limitation of imaging in the intracranial arteries. Although the plaque burden of intracranial artery is greater in Asian population, little data are available regarding the relationship between Hcy levels and intracranial plaque stability.

Currently, the application of high-resolution magnetic resonance imaging (HR-MRI) enables us to evaluate intracranial vasculopathy. Plaque characteristics on HR-MRI such as plaque enhancement and T1 hyperintensity can serve as markers of its stability and may associate with ischemic stroke [14–16]. In the present study, we aimed to evaluate the association between Hcy levels and intracranial plaque characteristics assessed on HR-MRI and investigated their clinical relevance in ischemic stroke events.

## Methods

### Subjects

This retrospective study was reviewed and approved by our institutional Review Board. The need for patient consent was waived by the same ethics committee. Between May 2016 and November 2017, we reviewed 146 consecutive patients with intracranial vasculopathy who had HR-MRI and, on luminal imaging, had intracranial arterial stenosis in our single institutional database. The detailed inclusion criteria were as follows: (1) plaque of MCA or basilar artery (BA) confirmed on HR-MRI; (2) absence of significant stenosis of extracranial carotid and vertebral artery (≥ 30%); (3) patients had one or more traditional atherosclerotic risk factors which included hypertension, vdiabetes mellitus, hypercholesterolemia and current cigarette smoking; and (4) the image quality of HR-MRI was adequate for evaluation. The exclusion criteria included: (1) non-atherosclerotic vasculopathies, such as vasculitis, dissection, or moyamoya disease; (2) patients had high risk factors for cardio-embolism, such as atrial fibrillation, valvular heart disease and infective endocarditis. Twelve patients with moyamoya diseases, 5 with arterial dissections, 2 with vasculitis, 10 with significant extracranial arterial stenosis, 7 with plaques only located in anterior cerebral artery or posterior cerebral artery, 7 with high-risk factors for cardio-embolism, and 9 with obvious motion artifacts on HR-MRI were excluded. Finally, 94 patients were recruited in the current study.

All the patients were divided into two groups based on the clinical information and diffusion-weighted imaging (DWI): 1) symptomatic group: patients who suffered from acute cerebral infarction or transient ischemic attack (TIA) in the distribution of MCA or BA territory within the recent 2 weeks; 2) asymptomatic group: patients with MCA or BA stenosis referred by luminal imaging before HR-MRI, yet had no history of stroke events.

### Clinical profiles and laboratory measurement

Laboratory examination including plasma Hcy levels, serum creatinine levels, total cholesterol, low-density lipoprotein and blood glucose were measured within 24 h of admission. Blood samples were drawn from the patients after overnight fasting. Venous blood samples were collected in tubes containing EDTA, centrifuged within 30 min to avoid a false elevation in Hcy concentration. The plasma Hcy levels were determined by high-performance liquid chromatography. The physiological range of plasma Hcy are 5–15 μmol/L. Patients with Hcy levels above 15 μmol/L were defined as high Hcy (hyperhomocysteinemia) [7].

The traditional atherosclerotic risk factors were defined as follows: (1) hypertension: blood pressure > 140/90 mmHg on repeated measurements, or patients receiving antihypertensive medication; (2) hyperlipidemia: total cholesterol level ≥ 200 mg/dL, low-density lipoprotein ≥130 mg/dL, or use of lipid-lowering agent; (3) diabetes mellitus: fasting blood sugar ≥7.0 mmol/L, or 2-h post prandial blood sugar ≥11.1 mmol/L, or use of antidiabetic medication. Stress hyperglycaemia was carefully excluded according to the hemoglobin A1c levels; (4) current cigarette smoker.

### MRI protocols

All the images were obtained with a 3.0 T MR imaging scanner (Magnetom skyra, Siemens Healthcare, Erlangen, Germany) equipped with a 20-channel head-neck coil. The HR-MRI protocols included a three-dimensional (3D) time-of-flight magnetic resonance angiography (TOF-MRA) and a 3D T1 weighted SPACE (Sampling Perfection with Application optimized Contrast using different angle Evolutions) sequence with fat-suppression before and after contrast administration. The detailed scan parameters used for T1 weighted SPACE were as follows: TR/TE, 900/4.2 ms; field-of-view, 240 × 216 mm; turbo-spin factor, 43 echos; echo spacing 4.2 ms. The acquired voxel size was 0.75 × 0.75 × 0.75 mm^3^ and reconstructed to 0.4 × 0.4 × 0.75 mm^3^. Contrast-enhanced T1 weighted SPACE was acquired with an approximately 5-min delay after administration of 0.1 mmol/kg contrast agent (gadodiamide, GE Healthcare, Ireland). Axial DWI was performed using a single-shot echo-planar spin-echo sequence with the following parameters: b-values, 0 and 1000 mm^2^/s, FOV, 230 × 230 mm, section thickness, 5 mm, matrix, 192 × 192.

### Image analysis

Two neuroradiologists (SSL and CQS, with 6 and 4 years of experience, respectively), who were provided each patient’s clinical presentation, DWI and TOF-MRA results, analyzed the HR-MRI images independently. A plaque was identified as focal wall thickening when it was evident on both the short and long axis of the vessel compared to the normal vessel wall. A culprit plaque was defined as the only or a most stenotic lesion within the vascular territory of the ischemic stroke. Plaque characteristics evaluated in our study included the following: (1) strong enhancement, enhancement was greater than or equal to that of pituitary stalk [14]; (2) T2 hyperintense components: presence or absence of a T2 hyperintense band adjacent to the lumen, which may indicate the fibrous cap [17]. Lesion signal intensity on T2 was assessed relative to gray matter; (3) T1 hyperintense components: presence or absence of T1 hyperintense on unenhanced T1 images with fat-suppression, which may indicate the intraplaque hemorrhage [16]. Lesion signal intensity on T1 was assessed relative to normal-appearing white matter. For any discrepancy between the two readers, another senior neuroradiologist (XNH with 20 years of experience) re-evaluated the images and assisted in reaching a consensus agreement.

The degree of luminal stenosis was calculated using the following formula: stenosis ratio = (1- narrow lumen diameter/reference lumen diameter) × 100%. The reference lumen was defined as the neighboring segment of normal appearance proximal to the stenotic site. The measurement results of luminal stenosis ratio from the two neuroradiologists were averaged for subsequent analysis.

### Statistical analysis

The inter-reader reproducibility for assessment of plaque characteristics was evaluated using Cohen’s kappa statistics. The intraclass correlation coefficient were calculated for measurements of luminal stenosis ratio. In the univariate analyses comparing between the symptomatic and asymptomatic group, plaque characteristics, luminal stenosis ratio, Hcy and serum creatinine levels, patient demographics (age, sex) and atherosclerotic risk factors were compared using a Student’s *t* test, or a Pearson’s chi-square test, or a nonparametric Mann-Whitney *U*-test as appropriate. Correlation of Hcy levels (normal or high Hcy) with plaque characteristics were examined by Spearman correlation analysis. Multivariable logistic regression was constructed to evaluate the association between high Hcy levels and plaque characteristics reaching significance on univariate analysis (model 1), and their ability to stratify the likelihood of ischemic stroke after adjusting for demographics, serum creatinine levels and vascular risk factors (model 2). Odds ratio (OR) with 95% confidence interval (CI) was calculated for the covariate with statistical significance. Receiver operating characteristic (ROC) curves were constructed to assess the diagnostic performance of high Hcy levels, significant plaque characteristics and their combination (model 2) to determine the occurrence of ischemic stroke. The ROC analysis was performed using MedCalc (version 12.3.0, Mariakerke, Belgium). All other statistical analyses were performed using SPSS (version 16.0, Chicago, IL, USA). Statistical significance was set as *P* < 0.05.

## Results

### Patient demographics and plaque characteristics

A total of 94 patients with ICAS were enrolled in the study. They were divided into two groups, symptomatic group (males = 54; females = 21; average age = 59.7 ± 12.8; 71 with DWI-evidenced stroke and 4 with TIA) and asymptomatic group (males = 14; females = 5; average age = 62.3 ± 16.6). For 4 patients who had a matched clinical presentation of TIA, the vascular distributions were as follows: left MCA (aphasia and numbness and weakness of the right leg; aphasia and decreased sensation in right arm), right MCA (left face and arm numbness and weakness) and vertebrobasilar artery (transient dysphagia). For 19 asymptomatic patients, 11 patients had not any symptom, 3 with headache, 3 with memory decline, and 2 with dizziness.

The plasma Hcy levels were 16.78 ± 5.52 μmol/L in symptomatic group, significantly higher than that of asymptomatic group (13.63 ± 5.01 μmol/L) (*P* = 0.026). The inter-reader agreement was 0.820 for strong plaque enhancement, 0.745 for T2 hyperintense components, 0.724 for T1 hyperintense components and 0.965 for stenosis ratio. More plaques in symptomatic group showed strong enhancement and presence of T1 hyperintense compared to that of asymptomatic group (*P* < 0.001 and *P* = 0.020, respectively). No significant difference was found in other plaque features. The baseline patients’ demographics and plaque characteristics are shown in Table 1.

**Table 1:** Patient demographics and plaque characteristics

Variables	Symptomatic group (*n* = 75)	Asymptomatic group (*n* = 19)	*P* value
Male, n (%)	54 (72.0%)	14 (73.7%)	0.883
Age, (Mean ± SD), y	59.7 ± 12.8	62.3 ± 16.6	0.454
Hypertension, n (%)	53 (70.7%)	16 (84.2%)	0.233
Diabetes mellitus, n (%)	28 (37.3%)	9 (47.4%)	0.424
Hyperlipidemia, n (%)	35 (46.7%)	10 (52.6%)	0.642
Current smoker, n (%)	24 (32.0%)	4 (21.1%)	0.351
Serum creatinine (μmol/L)	69.84 ± 16.31	71.32 ± 11.97	0.712
Homocysteine, (μmol/L)^a^	16.78 ± 5.52	13.63 ± 5.01	0.026
High Hcy, n (%)	48 (64.0%)	4 (21.1%)	0.001
Normal Hcy, n (%)	27 (36.0%)	15 (78.9%)	0.001
Plaque characteristics^b^			
Strong enhancement	54 (72.0%)	4 (21.1%)	< 0.001
Presence of T1 hyperintense	29 (38.7%)	2 (10.5%)	0.020
Presence of T2 hyperintense	38 (50.7%)	8 (42.1%)	0.505
Stenosis ratio (%)	61.7 ± 31.3	52.3 ± 30.0	0.303

Note: ^a^ The normal range of plasma Hcy is 5–15 μmol/L. High Hcy is defined as Hcy levels above 15 μmol/L. ^b^ Strong plaque enhancement, enhancement was greater than or equal to that of pituitary stalk; lesion T2 hyperintense components: presence or absence of T2 hyperintense band adjacent to the lumen; lesion T1 hyperintense components: presence or absence of T1 hyperintense on unenhanced T1 images with fat-suppression

### Association between high Hcy levels and plaque characteristics

Table 2 shows the distribution of plaque characteristics in relation to plasma Hcy levels. Strong plaque enhancement was observed in 41 of 52 (78.8%) patients with high Hcy levels and significantly correlated with high Hcy (*r* = 0.392, *P* < 0.001). To further examine the association of strong plaque enhancement with high Hcy, binary logistic regression analysis was performed (model 1). High Hcy was found to be associated with strong plaque enhancement independently of age, sex, serum creatinine levels and other traditional atherosclerotic risk factors (*P* < 0.001, OR 6.00, 95% CI 2.28–15.74) (Table 3).

**Table 2:** Plasma homocysteine levels and intracranial plaque characteristics

Plaque characteristics^b^	High Hcy^a^	Normal Hcy	*P* value
Strong enhancement	41 (78.8%)	17 (40.5%)	< 0.001
Presence of T1 hyperintense	19 (36.5%)	12 (28.6%)	0.414
Presence of T2 hyperintense	24 (46.2%)	22 (52.4%)	0.548
Stenosis ratio (%)	61.3 ± 31.7	58.0 ± 30.6	0.684

Note: ^a^ High Hcy is defined as Hcy levels above 15 μmol/L. ^b^ Strong plaque enhancement, enhancement was greater than or equal to that of pituitary stalk; lesion T2 hyperintense components: presence or absence of T2 hyperintense band adjacent to the lumen; lesion T1 hyperintense components: presence or absence of T1 hyperintense on unenhanced T1 images with fat-suppression

**Table 3:** Determinants of strong plaque enhancement (model 1)

Variables	B	OR	95% CI	*P* value
High Hcy^a^	1.791	6.00	2.28–15.74	< 0.001
Sex (male = 1)	0.041	1.04	0.30–3.66	0.949
Age (/y)	−0.006	0.99	0.96–1.03	0.764
Hypertension	−0.430	0.65	0.21–1.98	0.448
Diabetes mellitus	−0.108	0.90	0.33–2.44	0.832
Hyperlipidemia	−0.565	0.57	0.21–1.54	0.266
Current smoker	−0.234	0.79	0.26–2.44	0.685
Serum creatinine	0.002	1.00	0.97–1.04	0.934

Note: *OR* indicates odds ratio, *CI* confidence interval. ^a^ High Hcy is defined as homocysteine level above 15 μmol/L

### Ability of high Hcy and plaque characteristics to stratify ischemic stroke

**Fig. 1:**
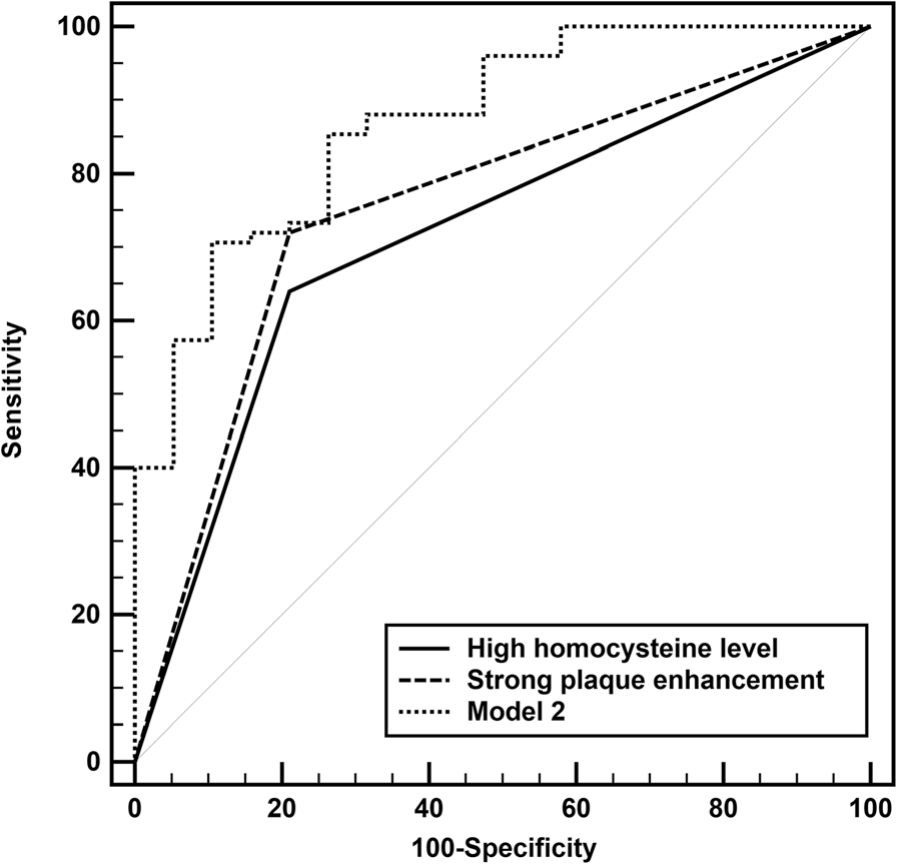
ROC curves for the ability of strong plaque enhancement and high homocysteine (Hcy) level to determine ischemic stroke. The AUC was 0.755 and 0.715 for strong plaque enhancement and high Hcy level, respectively. The combination of them after adjusting for sex, age, serum creatinine levels and other traditional atherosclerotic risk factors (hypertension, diabetes mellitus, hyperlipidemia, current smoker) in model 2 significantly improved the AUC to 0.871

In the multivariable logistic model (model 2), strong plaque enhancement (*P* = 0.026, OR 5.63, 95% CI 1.23–25.81) and high Hcy level (*P* = 0.018, OR 6.20, 95% CI 1.36–28.26) were associated with acute ischemic stroke independently of age, sex, serum creatinine levels and other traditional atherosclerotic risk factors (Table 4). The AUC (95% CI) for determining the occurrence of ischemic stroke were 0.755 (0.655–0.838) for strong plaque enhancement and 0.715 (0.612–0.803) for high Hcy level, respectively. The combination of high Hcy and strong plaque enhancement in model 2 improved the ability to stratify the likelihood for acute ischemic stroke, with improved AUC of 0.871 (0.786–0.931), significantly higher than that of strong plaque enhancement and high Hcy alone (*P* < 0.05 for both) (Fig. 1).

**Table 4:** Logistic regression analysis for predicting acute ischemic stroke (model 2)

Variables	B	OR	95% CI	*P* value
High Hcy^a^	1.824	6.20	1.36–28.26	0.018
Strong plaque enhancement	1.728	5.63	1.23–25.81	0.026
Presence of T1 hyperintense	1.748	5.74	0.81–40.77	0.080
Sex (male = 1)	0.381	1.46	0.26–8.37	0.668
Age (/y)	−0.015	0.99	0.94–1.04	0.559
Hypertension	−1.257	0.29	0.05–1.54	0.145
Diabetes mellitus	−1.069	0.34	0.08–1.50	0.155
Hyperlipidemia	0.287	1.33	0.32–5.52	0.692
Current smoker	0.799	2.22	0.45–10.90	0.324
Serum creatinine	−0.041	0.96	0.91–1.01	0.132

Note: *OR* indicates odds ratio, *CI* confidence interval. ^a^ High Hcy is defined as homocysteine level above 15 μmol/L

## Discussion

In the present study, we demonstrated a significant association between high plasma Hcy level and intracranial strong plaque enhancement independently of age, sex, serum creatinine levels and other traditional atherosclerotic risk factors in patients with large artery atherosclerosis. In addition, the combination of high Hcy and strong plaque enhancement improved the ability to stratify the likelihood for ischemic stroke in this group of patients.

Several plaque characteristics on HR-MRI were evaluated in our study. Plaque T1 hyperintensity is suggested as a possible indicator of recent intraplaque hemorrhage. The prevalence of T1 hyperintensity in our study was higher in plaques of symptomatic patients compared to those who were asymptomatic, which was consistent with previous study [16]. However, no correlation between high Hcy level and presence of T1 hyperintensity was found. Strong plaque enhancement may be the most promising imaging marker of intracranial plaque stability. In many previous reports, plaque enhancement has been documented as a very important predictor of plaque vulnerability in both extracranial and intracranial arteries [14, 15, 18–20]. A meta-analysis has shown that MRI-detected intracranial plaque enhancement is strongly associated with acute ischemic stroke [15]. In our study, plaques with strong enhancement was observed in 72% of plaques in symptomatic group, which is in accordance with previous findings. Pathologically, inflammation, neovascularity and increased endothelial permeability may contribute to the contrast enhancement of vulnerable plaques [21]. Plaques with strong enhancement reflects active inflammatory response and may lead to the occurrence of downstream cerebral infarction.

High plasma Hcy levels appeared to have association with intracranial strong plaque enhancement in our study. Previous studies have revealed that high Hcy may represent a sensitive risk factor for the development of advanced and unstable carotid plaques [7, 13]. Higher level of plasma Hcy were correlated with increased intima-media thickness in carotid artery [10, 22]. Our finding extends this association from carotid artery atherosclerosis to ICAS, further strengthening the existing evidence that elevated plasma Hcy level is a modifiable risk factor for vascular atherosclerosis. Hcy may impact the development of atherosclerosis through its involvement in complex pathways of inflammation and calcification [23]. It is postulated that high plasma Hcy levels may cause the impairment of vascular smooth muscle cell migration and endothelial cell function via the activation of methylation-sensitive transcription factors. It may also cause abnormalities in the function of fibrinogen and thrombin generation, leading to the formation of atherosclerotic plaques [24].

Elevated plasma Hcy level has been proved as an independent cardiovascular risk factor for ischemic stroke. Wu et al. found a significant correlation between elevated plasma Hcy and a greater incidence of acute cerebral infarction in patients with carotid artery plaques [7]. A prospective cohort study of Ji et al. supported the atherogenic role of Hcy. An elevated Hcy level can represent a sensitive predictor for severe neurological impairment, a poor functional outcome, and stroke recurrence in large artery atherosclerosis stroke subtype [25]. Several previous cohort studies such as the Northern Manhattan cohort study, the Framingham Study and the British Regional Heart Study also demonstrate elevated plasma Hcy was associated with an increased risk of ischemic stroke [26–28]. Our findings support this association. Both high Hcy levels and strongly enhanced plaques were significantly associated with symptomatic ischemic stroke. Importantly, the combined measurements of them were found to increase the ability to predict ischemic stroke events independently of sex, age, serum creatinine levels and other atherosclerotic risk factors. Such finding was consistent with a previous published study that the combination of carotid intima-media thickness and plasma Hcy levels could improve the prediction of ischemic stroke events [22]. High plasma Hcy is linked to mechanisms involved in the development of atherosclerosis, which may explain the synergism.

Our present study has certain limitations. First, homocysteine level is influenced by several factors which were not considered in this study, such as dietary habits (alcohol, coffee consumption, et al), folate and vitamin B_12_ levels, and therefore may potentially cause the bias. Second, this was a retrospective study, the selection bias cannot be discounted. The sample size was from a single Chinese center and relatively small, which may not be an accurate representation for all stroke patients, particularly in Western populations. Third, plaque characteristics in the present study were visually assessed. Quantitative analysis of plaque features, such as plaque burden and volume may provide incremental information on vulnerable lesions. Nevertheless, we consider that plaque enhancement is a non-invasive, sensitive and simple imaging marker in clinical practice. Finally, a longer period of following-up would probably allow us to examine the impact of plaque characteristics and/or Hcy levels on functional status (e.g., hospitalization duration and modified Rankin Scale), which is not discussed in the present study, but is clinically significant and deserve further investigation.

## Conclusions

Our data revealed a significant association between high plasma Hcy levels and strong plaque enhancement on HR-MRI. The combined assessment of plasma Hcy levels and plaque enhancement may improve ischemic stroke risk stratification.

## Abbreviations

Hcy: Plasma homocysteine
HR-MRI: High-resolution magnetic resonance imaging
ICAS: Intracranial atherosclerosis
MCA: Middle cerebral artery; BA, basilar artery
